# MgO:Li,Ce,Sm as a high-sensitivity material for Optically Stimulated Luminescence dosimetry

**DOI:** 10.1038/srep24348

**Published:** 2016-04-14

**Authors:** Luiz C. Oliveira, Eduardo G. Yukihara, Oswaldo Baffa

**Affiliations:** 1 Departamento de Física, FFCLRP-Universidade de São Paulo, 14040-901 Ribeirão Preto-SP, Brazil; 2 Physics Department, Oklahoma State University, Stillwater OK 74078, USA

## Abstract

The goal of this work was to investigate the relevant dosimetric and luminescent properties of MgO:Li_3%_,Ce_0.03%_,Sm_0.03%_, a newly-developed, high sensitivity Optically Stimulated Luminescence (OSL) material of low effective atomic number (*Z*_eff_ = 10.8) and potential interest for medical and personal dosimetry. We characterized the thermoluminescence (TL), OSL, radioluminescence (RL), and OSL emission spectrum of this new material and carried out a preliminary investigation on the OSL signal stability. MgO:Li,Ce,Sm has a main TL peak at ~180 °C (at a heating rate of 5 °C/s) associated with Ce^3+^ and Sm^3+^ emission. The results indicate that the infrared (870 nm) stimulated OSL from MgO:Li,Ce,Sm has suitable properties for dosimetry, including high sensitivity to ionizing radiation (20 times that of Al_2_O_3_:C, under the measurement conditions) and wide dynamic range (7 μGy–30 Gy). The OSL associated with Ce^3+^ emission is correlated with a dominant, practically isolated peak at 180 °C. Fading of ~15% was observed in the first hour, probably due to shallow traps, followed by subsequent fading of 6–7% over the next 35 days. These properties, together with the characteristically fast luminescence from Ce^3+^, make this material also a strong candidate for 2D OSL dose mapping.

Optically Stimulated Luminescence (OSL) technique is becoming a widespread method for personal and medical dosimetry since the development of Al_2_O_3_:C^1^,^2^. The all-optical nature of the OSL technique offers interesting advantages over the Thermoluminescence (TL) technique and explains the increasing acceptance of OSL dosimetry^2^. Nevertheless, the availability of only a few commercial materials for OSL dosimetry has been pointed out as a disadvantage of the OSL over the TL technique^3^, for which a wider range of materials is available^4^ . Al_2_O_3_:C and BeO remain the only two materials commercially used for OSL dosimetry^5^. Neutron and 2D dosimetry are areas that could benefit from new OSL materials having, for example, intrinsic neutron sensitivity (i.e. having ^6^Li or ^10^B in its matrix) or fast luminescence centers^6^,^7^.

The limited availability of OSL materials is critical for two-dimensional dose mapping in medical dosimetry (2D dosimetry), e.g., in quality assurance and dose verification of intensity modulated radiation therapy (IMRT). In 2D dosimetry, the OSL material must not only have suitable dosimetric properties, as Al_2_O_3_:C, but also fast luminescence centers (~μs) when using laser scanning readout, otherwise correction algorithms are required^8^,^9^. The problem is even more challenging if one considers the fact that, to manufacture an image plate, ideally an almost spherical polyhedron morphology with some distribution in size of the phosphor is required to ensure optimal packing^8^. Smaller particles will lose emission intensity via internal scattering, whereas larger particles make it difficult to produce thin films.

Although 2D dosimetry is possible using TL^10^,^11^, it is more easily achievable using an all-optical technique, as demonstrated by the use of the OSL in computed radiography^12^. The OSL (or photostimulable) phosphors used in computed radiography, such as BaXBr (X = F, Cl, Br) and CsBr, could be used, in principle, for 2D dosimetry, if not for their high effective atomic number (Z_eff_ > 49) and signal fading (>50% in 36 h)^12^.

One-dimensional dose mapping using Al_2_O_3_:C has been demonstrated and used commercially in computed tomography^13^, but the luminescence lifetime of ~35 ms from the main luminescence centers in this material (F-centers) is too long for 2D dosimetry readout by spot-scanning laser. This problem has been overcome by detecting the OSL signal originated from the fast (~7 ns) F^+^-center present in Al_2_O_3_ samples^14^ and using an algorithm to correct for the pixel-bleeding caused by the F-center emission^9^. Nevertheless, the development of a dosimetric OSL material which does not require the so-called “pixel-bleeding correction”, caused by the long luminescence lifetime of the F-centers in Al_2_O_3_:C, could further simplify the development of this technology.

Many studies have searched for new OSL materials with suitable properties for dosimetry, including halides (KCl, KBr, NaCl, RbI, CaF_2_, BaFX with X = Br, Cl, I), sulphates (MgSO_4_, CaSO_4_), sulphides (AS, with S = Mg, Sr, Ca, Ba) and oxides (BeO and fused quartz)^2^. These materials, however, have not been adopted in dosimetry, with the exception of BeO^15^,^16^,^17^, probably because most of them showed one or more undesirable features such as fading, low sensitivity to radiation, high effective atomic number, or even self-dose. For example, in spite of their near tissue equivalence, Mg_2_SiO_4_:Tb, LiAlO_2_:Tb, and other Tb-activated silicates and aluminates were found to have much lower sensitivity than Al_2_O_3_:C^18^. YAlO_3_:Mn^19^ and Y_3_Al_5_O_12_:C^20^ were found to be very sensitive, but they have high effective atomic number (*Z*_eff_ = 32.3 and 30.6, respectively). KMgF:Ce also exhibit OSL sensitivity about 10 times higher than that of commercial Al_2_O_3_:C when stimulation by blue LED (470 nm)^21^, but it suffers from a high self-dose (~1.5 μGy/h), due to the natural abundance of ^40^K, and is not tissue equivalent (Z_eff_ = 14.7). NaMgF:Ce has low effective atomic (Z_eff_ = 11.0) and OSL sensitivity comparable to that of Al_2_O_3_:C using blue stimulation, but exhibits severe fading^21^. KBr:Eu and KCl:Eu have drawn attention for two-dimensional imaging in medical diagnostics and 2D dose mapping in radiotherapy^22^,^23^,^24^,^25^,^26^, but their effective atomic numbers are also relatively large (Z_eff_ = 18.1 for KCl:Eu and Z_eff_ = 31.5 for KBr:Eu).

MgO is an interesting material for 2D dosimetry: it has a low effective atomic number (Z_eff_ = 10.8), simple cubic lattice structure^27^, and many defects have been investigated by optical absorption, electron paramagnetic resonance, and TL, among others^28^. Due to its high chemical stability (melting point of 2800 °C) and wide band gap (7.8 eV^29^), it opens up the possibility of engineering a large variety of stable trapping and color centers.

Recent investigations of OSL in MgO showed that TL and OSL can be enhanced or modified by lanthanide (Ln) doping^30^,^31^, allowing control of the OSL properties by an appropriate choice of dopant^30^,^31^,^32^. Doping with Ln is interesting because it is possible, in principle, to estimate the energy level for each Ln inside the energy band diagram, provided that the energy of at least one Ln of a given valence is known^33^. This is possible because the energy differences between Ln dopants of the same valence are constant regardless of the matrix. Thus, knowledge of the energy level with respect to the top of the valence band or to the bottom of the conduction band can indicate, beforehand, whether the Ln will act as an electron trap (acceptor) or as a hole trap (donor). This model was successful in explaining the several TL peaks on YPO_4_:Ce co-doped with another trivalent Ln^34^. It is worth noting that the YPO_4_ matrix represents a special case, since the trivalent Ln would replace the Y-site without need for charge compensation. Nevertheless, for a non-trivalent site, charge compensation would be required for a trivalent Ln to substitute the metal site. In this work, MgO was doped with Ce and Sm, but since MgO is a divalent matrix, the substitution of Mg-site by one trivalent Ln requires charge compensation, and for this this purpose, Li was used. It was demonstrated that Li co-doping can improve the TL intensity and the RL output for MgO doped with different Ln^31^,^32^,^35^.

The purpose of this work was to investigate the main dosimetric and luminescence properties of a new MgO formulation, MgO:Li_3%_,Ce_0.03%_,Sm_0.03%_, prepared by Solution Combustion Synthesis (SCS) for OSL dosimetry. Doping with 3% of Li was found to be the minimum required to enhance the OSL/TL signal intensity^31^,^32^. On the other hand, doping with Ce^3+^ and Sm^3+^ was intentionally small (0.03%) to avoid increasing the Z_eff_ of the material. The samples were characterized using TL, OSL, RL techniques. OSL and TL emission spectra were also obtained. The morphology of the phosphor was analyzed by scanning electron microscopy. We carried out an investigation of the OSL signal stability for different storage times at room temperature and also investigated the effect of precursor purity on TL and OSL signal intensity.

This work is part of an effort to develop new OSL materials for personal or medical dosimetry. The new detector is a result of a systematic investigation carried out on doped MgO samples designed to meet the requirements for two-dimension dosimetry for medical application. These requirements include low Z_eff_, high sensitivity to ionizing radiation, fast luminescence lifetime, wide dynamic range, linear dose response and signal stability. The optimum concentration and role of each dopant in the OSL mechanism are currently under investigation and will be reported in the future.

## Material and Method

### Synthesis and sample preparation

Polycrystalline MgO was synthesized by Solution Combustion Synthesis (SCS) by first forming aqueous stock solutions of suitable chemical reagents, i.e., magnesium nitrate hexahydrate (Mg (NO_3_)_2_.6H_2_O, ACS, 98–102%, Alfa-Aesar) and urea (CO(NH_2_)_2_, ACS, 98%, Alfa-Aesar) in distilled water of 2 M and 8.33 M, respectively. Undoped MgO samples were prepared by mixing 25 ml of magnesium nitrate solution of 2 M with 10 ml of urea solution of 8.33 M in a 400 ml beaker. The beaker was then placed on a hot plate and initially mixed using a magnetic stir bar for approximately 2 min at room temperature. The magnetic stir bar was then removed and the temperature of the hot plate was raised to 200 °C for water evaporation. After one hour at 200 °C, water evaporation comes to a completion and the hot plate temperature was again raised to 500 °C until reaction occurred, after ~8 min. After reaction completion, the obtained white solid paste was removed from the bottom of the beaker and ground with an agate mortar and pestle. The resulting powder was placed in an aluminum crucible for further annealing.

Doped MgO samples were prepared in a similar fashion, except that the desired dopant was added to the master solution. Thus, a solution of lithium nitrate (LiNO_3_, 99%, Alfa-Aesar), cerium nitrate (CeNO_3_)_3_.6H_2_O, ACS, 99,9%, Alfa-Aesar), and samarium nitrate (SmNO_3_)_3_.6H_2_O, ACS, 99,9%, Alfa-Aesar), or a combination of them were added to the master solution. Doped and undoped MgO samples were also synthesized using magnesium nitrate of high purity (Mg (NO_3_)_2_· xH_2_O, Puratronic^®^, 99,999, Alfa-Aesar,) as well as urea of higher purity (CO(NH_2_)_2_, ACS, 99.3%, Alfa-Aesar).

Annealing was performed in an electric furnace (Marshall model 1123, Thermcraft Inc.) in open atmosphere. The samples were placed in alumina crucibles and heated from room temperature to 900 °C or 1000 °C at 5 °C/min, held at that temperature for 2 hours, and allowed to cool down naturally afterwards. The powder was removed from the crucible and ground again in an agate mortar and pestle.

Throughout the text, we refer to the samples prepared using magnesium nitrate of 99.999% purity and urea of 99.3% purity as “high-purity” MgO, and to the samples prepared using magnesium nitrate of 98–103% purity and urea of 98% purity as “low-purity” MgO. The dopant precursors were the same for both cases. MgO:Li_3%_,Ce_0.03%_,Sm_0.03%_ annealed at 1000 °C for two hours is here designated MgO:Li,Ce,Sm. Other conditions will be mentioned explicitly. The crystalline phase was confirmed by X-ray diffraction measurement and presented earlier^32^.

### Detector preparation

Some TL and OSL measurements were carried with samples in powder form. For the measurements of dose response, OSL emission spectrum and fading, however, we prepared detectors consisting of MgO:Li,Ce,Sm powder encapsulated in plastic sheets. (Due to the plastic, no TL measurement was performed on these detectors.) The detectors present a disc-like shape with 7.20 mm diameter and 0.20 mm of thickness and are robust and easy to handle. This detector preparation also protected the powder from environmental humidity, assuring chemical stability and, consequently, the stored information.

**Figure 1:**
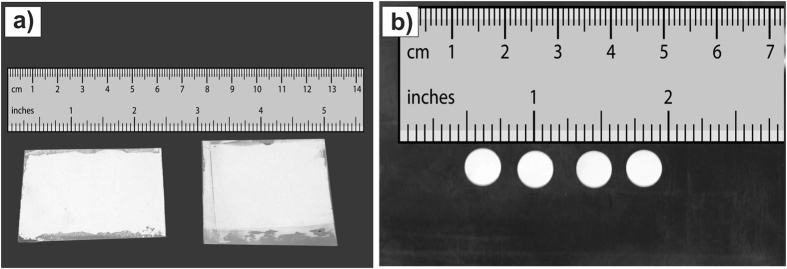
(**a**) Film prepared by depositing MgO:Li,Ce,Sm on transparent plastic sheets and (**b**) detectors after being cut in disc shape.

The detectors were prepared as follows. Fine powder of MgO:Li,Ce,Sm was thoroughly mixed with an organic resin, used as a binder, and homogeneously deposited in a transparent plastic sheet. The sheets were allowed to cure at 80 °C for several hours and then a transparent tape was used for cover the adhered powder. Figure 1a shows two sheets prepared as described above and in Fig. 1b the detector in disc shape after cutting.

### Characterization

TL measurements were carried out using an automated Risø TL/OSL-DA-15 reader (Risø National Laboratory, Røskilde, Denmark). OSL measurements were performed using either the Risø reader or a custom-built OSL reader. The choice of reader was just a matter of convenience: for investigations involving a large number of repetitions, we used the automated Risø reader system; otherwise, the custom-built was preferred for its prompt availability.

The Risø reader is equipped with a ^90^Sr/^90^Y beta source for irradiation and a photomultiplier tube (PMT) for light detection (model 9235QB, Electron Tubes, Inc., Middlesex, UK). TL measurements were performed at 5 °C.s^−1^ in nitrogen atmosphere and using a BG-39 filter for detection (6 mm total thickness, Schott AG, Mainz, Germany, transmission between 330 and 620 nm). The OSL readouts were carried out in the Risø using continuous-wave (CW) infrared light-emitting diodes (peak emission at 870 nm and irradiance of ~180 mW cm^−2^ at the sample position).

The OSL signal stability study was performed using the custom-built OSL reader, which is equipped with three infrared light-emitting diodes operating in continuous-wave mode (model M850D2, peak emission at 850 nm) for sample stimulation and a PMT (model 9250B04, Thorn-EMI electron tube) for photon detection. To block the stimulation light, two BG-39 bandpass filters (6 mm total thickness, Schott AG, transmission between 330 and 620 nm) and an OD 2 Shortpass filter (5 mm thickness, 575 nm cut-off wavelength, transmission 400–550 nm) were placed in front of the PMT. The PMT was connected to a photon-counting unit (model C3866, Hamamatsu). The photon-counting unit converts the PMT signal to TTL pulses through built-in amplifier and discriminator circuits. The referred unit uses a high-speed electronic circuit that allows measurement with output linearity up to 10^7 ^s^−1^. The TTL pulses were integrated using an electronic digital acquisition (DAQ) board. The equipment was controlled using LabView (National Instrument Corporation, Austin, TX, USA).

TL and OSL measurements were typically carried out using ~10 mg of powder deposited in stainless steel sample carriers, except for the fading test and the OSL emission spectrum measurement. For these experiments, the measurements were carried out using the prepared detectors (material deposited on a transparent plastic sheet). A commercial TL material (LiF:Mg,Ti powder, TLD-100, Thermo Fisher Scientific Inc.) and an OSL material (Al_2_O_3_:C powder, Landauer Stillwater Crystal Growth Division, Landauer Inc., Stillwater, OK, USA) were used for comparison.

The samples were irradiated inside the Risø reader using the ^90^Sr/^90^Y source, except for the fading experiment. In this case we used a 40 kV X-ray tube (40 kV Magnum™ X-ray tube, W transmission target, Moxtek Inc., Orem, UT, USA), delivering a dose rate of approximately 0.45 Gy.min^−1^ at the sample position, which was about 20 cm away from the head of the x-ray tube.

RL measurements were performed in a custom-built equipment consisting of an excitation source and a CCD spectrophotometer. The sample was excited with X-rays (the same X-ray tube as above) with a dose rate of approximately 5 Gy.min^−1^ at the sample position. The RL signal was collected using a f/2 fused silica lens coupled to an end of an optical fiber (1 mm core diameter, transmission between 200 and 1100 nm). The other end of the optical fiber was connected to a spectrometer (Ocean Optics USB-2000, Inc., Dunedin, FL, USA). The RL spectra were obtained using ~10 mg of powder placed in stainless steel cups. The wavelength response of the system is reported in ref. 32.

OSL emission spectrum and OSL stimulation spectrum were carried out using a spectrofluorometer (Fluorolog-3, model FL3-22, Horiba Instruments Incorporated, Edison, NJ, USA) at room temperature. The OSL emission spectrum was obtained by stimulating the irradiated samples with 700 nm light while scanning the detection from 300 nm to 650 nm in 1 nm steps and 0.1 s integration time. A longpass filter (OG-500 filter, 3 mm thickness, Schott AG) was placed in front of the stimulation light source to prevent UV light reaching the sample.

The sample’s morphology image was carried out using a FEI Quanta 600 field-emission Scanning Electron Microscope with magnification of 12000 and acceleration voltage of 10 kV. The image was performed on dried drops of the suspension, containing fine particles of MgO, on an aluminum stub and coated with gold palladium. The particles were suspended in ethanol by ultrasound bath.

## Results and Discussions

### Morphology

**Figure 2:**
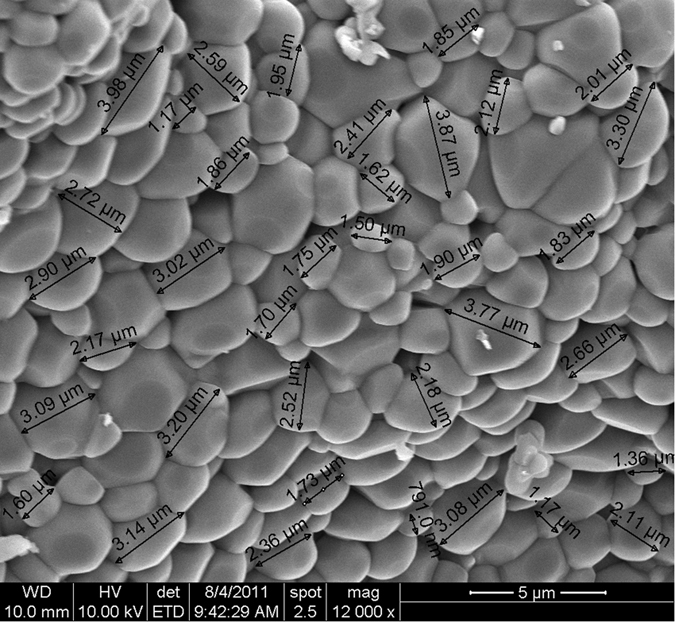
Scanning electron microscopy image of MgO particles annealed at 1100 °C for one hour. One can observe a regular morphology with almost spherical grains with size averaging from 1 to 4 micrometers.

Figure 2 shows the SEM image of MgO prepared by SCS. The morphology is characterized by regular crystallites with an average size of 1–4 micrometers, which is suitable for film casting^8^.

### Basic luminescence properties

**Figure 3:**
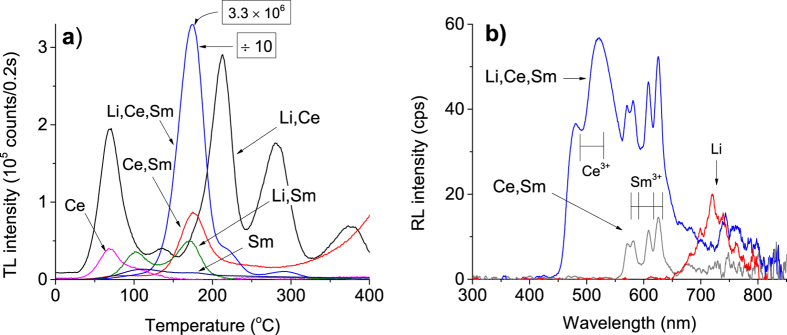
(**a**) TL curves of MgO: Ce_0.3%_, MgO:Li_3%_,Ce_0.3%_, MgO:Ce_0.3%_,Sm_0.3%_, MgO:Li_3%_, MgO:Li_3%_, Sm _0.3%_ and MgO:Li_3%_,Ce_0.3%_,Sm_0.3%_ (Schott BG-39 filters). The TL signal of MgO:Li_3%_,Ce_0.3%_,Sm_0.3%_ was reduced by a factor of 10 for comparison. (**b**) RL emission spectra of MgO:Li_3%_, MgO:Li_3%_,Ce_0.3%_,Sm_0.3%_ and MgO:Ce_0.3%_,Sm_0.3%_. Samples were synthesized with starting material of inferior purity and annealed at 900 °C for 2 h.

Figure 3a shows the TL curve of MgO samples prepared with different dopants, namely, MgO:Li_3%_, MgO:Sm_0.3%_, MgO:Ce_0.3%_, MgO:Li_3%_,Ce_0.3%_, MgO:Ce_0.3%_,Sm_0.3%_ and MgO:Li_3%_,Ce_0.3%_,Sm_0.3%_. The samples were synthesized using low-purity reagents and annealed at 900 °C for 2 h. Undoped MgO did not show any significant TL emission and was not included in Fig. 3a. The combination of Ce and Sm gave rise to a new TL curve, characterized by an almost isolated TL peak at 180 °C, that is different from the TL curves reported for other lanthanide-doped MgO samples^30^,^31^,^32^ or MgO doped with transitions metals^36^,^37^. (See also other TL curves for MgO in Las and Stoebe^28^. When comparing the TL curves from different studies, however, one should keep in mind that the TL curves and peak positions can be affected by the detection window and heating rate used in each study.) It was also found that 3% Li co-doping increases the TL peak intensity at least 30 times, making the TL of this material about 10 times more intense than the already sensitive MgO:Li,Ce (see Fig. 3a). The TL peaks of MgO:Li,Ce now appear as shoulders in MgO:Li,Ce,Sm sample (Fig. 3a). Notice that the TL curve intensity of MgO:Li,Ce,Sm shown in Fig. 3a was divided by a factor of 10, for better visualization.

Figure 3b shows the most significant RL emission spectra of doped MgO, namely, of MgO:Li_3%_, MgO:Ce_0.3%_,Sm_0.3%_ and MgO:Li_3%_, Ce_0.3%_,Sm_0.3_. In the MgO:Ce,Sm sample, the Ce emission is barely detected with our CCD spectrometer, but co-doping with Li enhances the Ce^3+^ emission several times. It is also clear that the band emission from 450 nm to 550 nm is not due to Li, since MgO doped with only Li does not show this emission (Fig. 3b). The hypothesis that these emission bands can be attributed to Ce^3+^ is corroborated by luminescence studies by Bapat *et al.* on MgO:Li,Ce samples^38^, where the authors reported a spectrum similar to that shown in Fig. 3b, characterized by two bands with a weaker emission peaking at 490 nm and main peak with maximum at 525 nm. Ce^3+^ emission is associated with 5d→4f transition and is typically characterized by a double band due to splitting of the 4f^1^ ground state into ^2^F_5/2_ and ^2^F_7/2_ states^8^. Although the luminescence lifetime of Ce^3+^ was not measured, Ce^3+^ is known to have fast luminescence lifetime (<100 ns) due to the allowed 5d-4f transition. In fact, the fast 15–60 ns 5d-4f emission of Ce^3+^ in compounds like Lu_2_SiO_5,_ LaCl_2_, Gd_2_SiO_5_, LaBr_3_ is utilized in scintillators for ϒ-ray detection (see^39^ and references therein). The four narrow emissions in the range between 550 nm and 650 nm in Fig. 3b are assigned to the ^4^G_7/2_→^6^H_9/2_, ^4^G_5/2_→^6^H_7/2_, ^4^G_5/2_→^6^H_9/2_ and ^4^G_7/2_→^6^H_11/2_ transitions of Sm^3+^, respectively, from left to right^40^.

**Figure 4:**
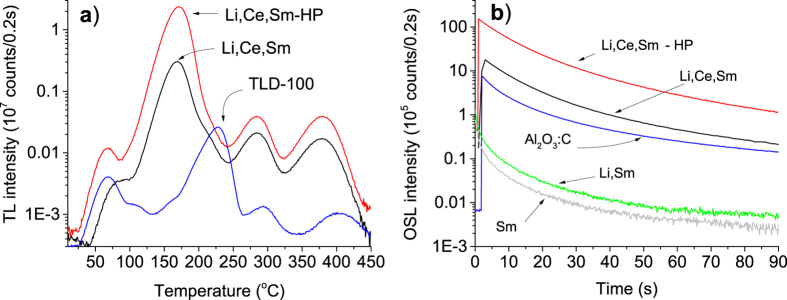
(**a**) TL (5 °C s^−1^) and (**b**) OSL curves of MgO:Li,Ce,Sm (IR-stimulation, Schott BG-39 filters) prepared with low and high-purity reagents, annealed at 1000 °C for 2 h and irradiated with doses of 0.5 Gy. The OSL curves of MgO:Li and MgO:Li,Sm prepared with reagents of low purity were also included for comparison. The curves are shown in log scale to better show the intensity of each TL curve. The TL and OSL signals of MgO prepared with high-purity reagents are about 8 times higher than those prepared using low-purity reagents. The TL of TLD-100 and the OSL of Al_2_O_3_:C (blue stimulation, Hoya U-340 filters) are also included for comparison.

We have found that the different concentrations of Ce and Sm, the purity of the starting material and the annealing temperature affect the intensity but not the shape of the TL curve. The TL curves of MgO:Li,Ce,Sm synthesized using either high or low purity reagents and annealed at 1000 °C are shown in Fig. 4a. The high-purity samples (HP) presented a TL signal about 8 times more intense than that of low-purity samples. The TL curve of TLD-100 is also presented for comparison. The intensity of the main TL peak of MgO:Li,Ce,Sm is about 80 times higher than the main TL peak of TLD-100. Nevertheless, one of the most remarkable features of the MgO:Li,Ce,Sm developed here is the low intensity of the low temperature TL peaks. Low temperature TL peaks are associated with signals that are unstable at room temperature. These shallow trapping centers can also contribute to a thermally unstable OSL signal that is difficult to separate from the main OSL signal of interest for dosimetry; this is not the case in TL curves, where the signals appear as separate TL peaks.

Figure 4b shows the OSL signal from the two MgO:Li,Ce,Sm prepared as described above, compared with the OSL signal from Al_2_O_3_:C. The measurements were carried out using the same mass of material but using IR stimulation and Schott BG-39 filters for detection in the case of MgO:Li,Ce,Sm, and blue stimulation and Hoya U-340 filters in the case of Al_2_O_3_:C.

Similarly, to the case of TL, the OSL signal intensity is higher in high-purity samples. The initial intensity of MgO:Li,Ce,Sm is about 20 times more intense than Al_2_O_3_:C, although the filter combination is not optimum for Al_2_O_3_:C, since the Hoya U-340 filters block a large portion of the F-center emission of Al_2_O_3_:C^41^. It is worth pointing out that not only the initial OSL intensity but also the total OSL of MgO:Li,Ce,Sm (area under the OSL curve) are higher than those of Al_2_O_3_:C.

In this study, we synthesized three batches using the same procedure and the results showed a standard deviation of only 10%, indicating a good repeatability of the synthesis procedure.

### TL and OSL signals correlation

TL and OSL were correlated by means of the so-called step-annealing procedure^42^. The process can be described as follows: (i) the sample was irradiated with ionizing radiation at room temperature; (ii) after irradiation, the sample was heated from room temperature up to a pre-defined temperature *T*_stop_; (iii) the sample was allowed to cool back to room temperature, and then (iv) the OSL was recorded. The whole process with the same sample with increasing *T*_stop_ in a chosen range is repeated, but before that, the sample was heated to 450 °C to clean the deep traps. For this experiment, a dose of ~0.5 Gy and a heating rate of 5 °C.s^−1^ were used. The OSL signal was recorded for 60 s stimulation time.

**Figure 5:**
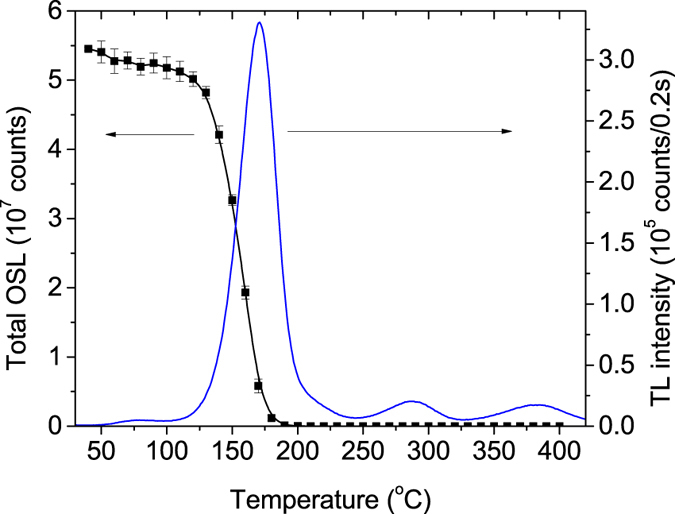
OSL and TL curves of MgO:Li,Ce,Sm after various pre-heating temperatures. Each OSL data point corresponds to the total area under the OSL curve after irradiation and pre-heating to the indicated temperature. The uncertainties were estimated based on the measurement of three aliquots. A dose of 0.5 Gy was used for each OSL measurements.

Figure 5 shows the step-annealing curve obtained in the range from 40 °C to 450 °C in 10 °C intervals. Each data point represents the mean value and standard deviation of the total OSL signal for three aliquots. This data indicates that the OSL signal arises mainly from the main TL peak at 180 °C. Shallow traps make a small contribution to the OSL signal, as observed by the initial portion of the OSL versus *T*_stop_ curve. On the other hand, the small TL peaks at higher temperature do not contribute to the OSL signal. This result demonstrates that the OSL signal comes from a relatively deep trap that gives rise to the 180 °C TL peak. The result also suggests that the mechanism involved in the generation of the OSL signal is relatively simple, the OSL originating mostly from a single trapping center, at least under the stimulation wavelength used in this measurement (870 nm).

### Emission spectrum

**Figure 6:**
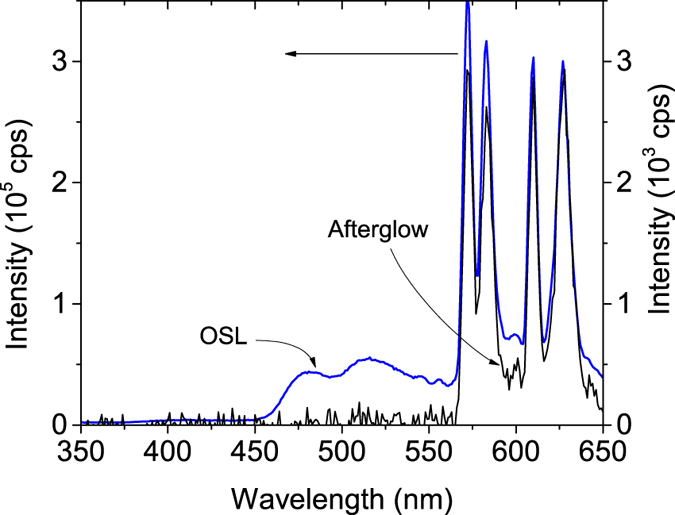
Emission spectra of MgO:Li,Ce,Sm recorded using two different conditions: (right y-axis) without stimulation (afterglow) and with (left y-axis) stimulation with 700 nm (OSL). The measurements were performed in a sample that has been previously exposed to ~30 Gy of x-ray source.

Figure 6 shows the phosphorescence spectrum (no light stimulation) and the OSL emission spectrum of MgO:Li,Ce,Sm (with light stimulation). The spectra were taken by scanning the emitted light of a previously x-ray irradiated sample in the 300–650 nm range. The OSL emission spectrum was taken by scanning the emitted light from the sample while the sample was continuously stimulated with a 700 nm light. For these measurements, a freshly irradiated sample with ~30 Gy was used.

One can clearly see the similarities of the OSL emission spectrum shown in Fig. 6 with the one RL spectra in Fig. 3b. Except for their relative intensities, the spectrum consists of ~480 nm and ~517 nm broad bands attributed to Ce^3+^ emission, and sharp peaks from 550 nm to 650 nm due to Sm^3+^. It is worth noticing that, whereas the afterglow spectrum consists of characteristic Sm^3+^ emissions lines only, the OSL spectrum shows both Ce^3+^ and Sm^3+^ emissions. The intensity of the afterglow spectrum is also two orders of magnitude weaker than the OSL emission spectrum.

**Figure 7:**
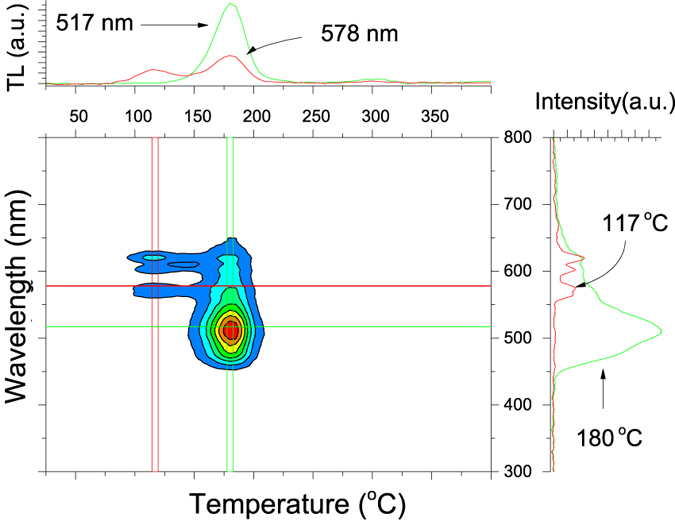
TL emission spectra of MgO:Li,Ce,Sm shown as a contour plot. The sample shows emission from two dopants, namely, Ce^3+^ and Sm^3+^. The temperature is given by the x-axis and wavelength by the y-axis. On the top, the TL curve is obtained by monitoring one of the main line from each dopant, i.e., Ce^3+^ (~517 nm) and Sm^3+^ (~578 nm, for example). In the right side of the counter plot, the TL spectrum are recorded monitoring different TL peaks, a namely, the 117 °C and the 180 °C, as indicated in the figure.

Figure 7 shows the TL emission spectrum of the MgO:Li,Ce,Sm sample in a contour graph. The sample shows the characteristics emissions from two dopants, namely Ce^3+^ and Sm^3+^. In the same figure, on the top, the TL curve is obtained by monitoring one of the main lines from each dopant, i.e., Ce^3+^ (~517 nm) and Sm^3+^ (~578 nm, for example). In the right side of the counter plot, the TL spectrum is recorded monitoring different TL peaks, the 117 °C and the 180 °C, as indicated in the figure.

By monitoring the Ce^3+^ emission one can note that the TL curve associated with Ce^3+^ does not exhibit a shallow trap. Actually, the TL curve is due to just one TL peak at 180 °C. Conversely, monitoring one of the Sm^3+^ emission lines leads to the conclusion that, besides the TL peak at 180 °C, a peak at 117 °C also is present. Monitoring the emission spectrum of the 180 °C one finds that the main emission is due to the Ce^3+^ spectrum, while monitoring the 117 °C peak one finds that it is due mainly to the Sm^3+^ emission lines (left side of figure).

### Dose response

**Figure 8:**
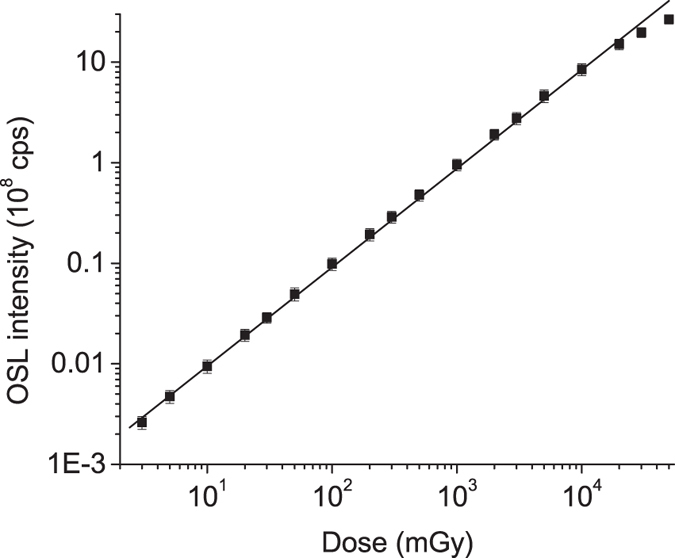
OSL dose response based on the initial OSL signal under infrared stimulation for a MgO:Li,Ce,Sm sample. Each data point is based on the measurement of three aliquots; the experimental uncertainties (experimental standard deviation) are represented by error bars (barely visible). The straight line indicates the linearity.

Figure 8 shows the dose response curve of MgO:Li,Ce,Sm. The measurements were carried out using three aliquots of 10 mg each. The OSL signal is linear from 2 mGy up to 20 Gy, after which the signal starts to saturate. The minimum detectable dose in the current experimental conditions was estimated by calculating the sample sensitivity (total OSL area divided by the dose) for three aliquots and determining the dose corresponding to three times the standard deviation of the background (3σ BG). The background was estimated by three measurements of an un-irradiated sample. In these experimental conditions, the minimum detectable dose was estimated to be ~7 μGy using the total OSL area and ~0.2 μGy using the initial OSL intensity. The low minimum detectable dose and linearity until >20 Gy demonstrates the wide OSL dynamic range of MgO:Li,Ce,Sm.

### Fading

**Figure 9:**
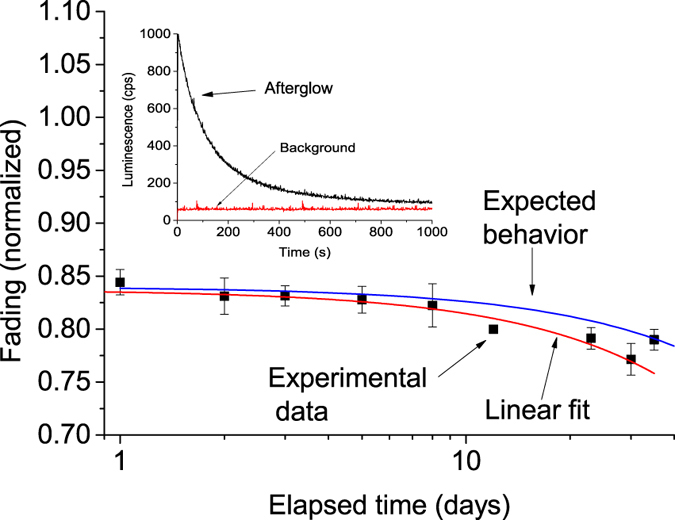
Effect of storage on the total OSL signal of MgO:Li,Ce,Sm measured after different storage times followed irradiation. Each data point is a result of measurement of three aliquots and the error bars represent the experimental standard deviation. The red curve is the linear fit to the experimental data (in linear scale). The blue curve represents the expected exponential fading behavior given by the expected mean lifetime *τ* of the main trap, where *τ*^−1^ = *p* = *s* exp(−*E/kT*), with *s* = 1.31×10^13 ^s^−1^, *E* = 1.22 eV, and *T* = 295 K (*k* is the Boltzmann constant). The inset figure shows the afterglow curve of up to 1000 seconds obtained immediately after irradiation.

The capability of a phosphor to store information until readout is of paramount importance in dosimetry. Figure 9 shows the signal fading behavior of the MgO:Li,Ce,Sm sample. The fading test was carried out by exposing several discs made of MgO:Li,Ce,Sm to 200 mGy of X-ray irradiation. The irradiated detectors were kept in a light tight drawer, at room temperature. After different storage times, three aliquots were taken for read out. The signal loss is given by the ratio between the OSL signal from the stored sample and the signal from the same disc read immediately after irradiation. The samples exhibited a strong fading in the first hours (~15%), followed by an OSL fading of about 6% to 7%, within 35 days of storage time (Fig. 9). The inset of Fig. 9 shows the afterglow of this sample after a dose of 0.5 Gy and immediately read, but without stimulation. One can see that the afterglow is strong, but decays exponentially, explaining the decrease in signal in the first hour after irradiation.

The expected fading behavior of the main TL peak is also presented in Fig. 9 for comparison (blue curve). This curve represents the expected mean lifetime *τ,* calculated using the kinetics parameters of the trap responsible for the 180 °C TL peak. Details of the calculation are discussed below. One can observe that the predicted fading is comparable to the experimental measurements, indicating that the fading can be explained by room temperature decay of the TL peak.

To better understanding the fading shown in Fig. 9, the expected mean lifetime *τ* of the charge carrier in the trap, responsible for the main TL peak, was calculated. This calculation was performed using the expression *τ* = *s*^−1^exp *(E/kT)*, where k is the Boltzmann constant in unit of eV.K^−1^ with *E* and *s* being the trap depth and frequency factor given in eV and s^−1^ units, respectively, and *T* is the storage temperature in Kelvin. The kinetics parameters, *E* and *s*, were calculated using the peak position-heating rate method^43^. The plot of ln(*T*_*m*_^2^*/β*) against *1/kT*_*m*_ using the expression *βE/kT*_*m*_^2^ = *s* exp(−*E/kT*_*m*_), where *β* is the heating rate and *T*_*m*_ is the position of the peak, gives a straight line of slope *E*. From the intercept, one can calculate the frequency factor *s*. It is worth pointing out, however, that this particular equation is only valid for TL peak of first-order kinetics.

**Figure 10:**
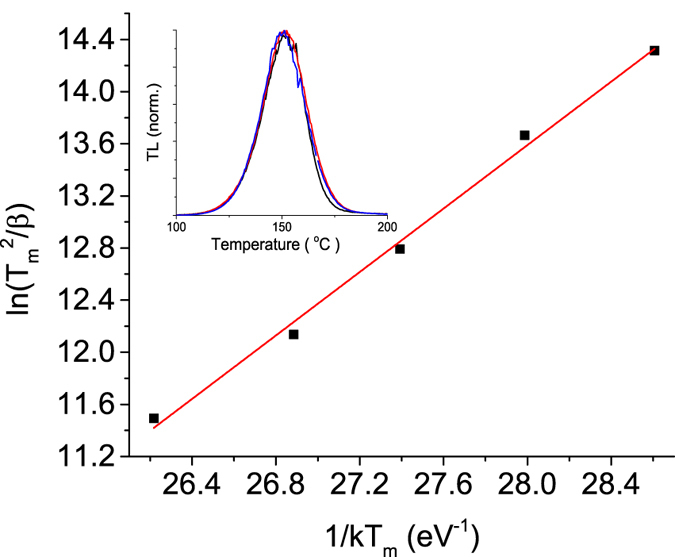
Plot of ln(T_m_^2^/β) against 1/kT_m_ for MgO:Li,Ce,Sm. The measurements were carried out with heating rates of 0.1, 0.2, 0.5, 1 and 2 °C.s^−1^. The inset shows the behavior of the normalized TL peak after x-ray exposures of 2 s, 10 s and 100 s. The TL was measured with a heating rate of 1 °C.s^−1^.

Figure 10 shows the heating rate plot of ln*(T*_*m*_^2^*/β)* against *1/kT*_*m*_ for MgO:Li,Ce,Sm. The measurements were carried out with heating rates of 0.1, 0.2, 0.5, 1 and 2 °Cs^−1^. Through the fitting parameters we obtained the energy trap depth *E* = (1.22 ± 0.05) eV and frequency factor s = 1.31×10^13 ^s^−1^. This activation energy is of the order of the predicted value of (1.6 ± 0.5) eV according to Dorenbos *et al.* (see ref. 44 and ref. therein) using data of charge transfer spectroscopy for MgO:Eu^45^, which supports the hypothesis Sm^3+^ is acting as an electron trap. Normally, *s* values are expected to be of the order of the Debye frequency, namely 10^12^ − 10^14 ^s^−1^. The inset in Fig. 10 shows the TL peak behavior after exposure to x-ray for 2 s, 10 s and 100 s, or approximately, 200 mGy, 1 Gy and 10 Gy. One interesting thing to note is that the peak shape and the peak position are unaffected by different trap populations. This is characteristic of a first-order kinetics TL peak.

Using the values obtained above for the frequency factor (*s* = 1.31 × 10^13 ^s^−1^) and activation energy (*E* = 1.22 eV), as well as assuming first-order kinetics and a storage temperature of 22 °C, the lifetime can be estimated by *τ* = *s*^−1^exp *(E/kT)*, which gives approximately ~1.7 years. With this lifetime, the predicted fading at 22 °C in 35 days is approximately 5.5%, which agrees with the experimental data in Fig. 9. Thus, the observed fading is compatible with trap kinetics. We cannot exclude the possibility of fading caused by the room red LED (~650 nm) light used during sample handling, but we used dim light when manipulating the samples.

## Discussion

Possible models for the TL, OSL and luminescence behavior of MgO:Li,Ce,Sm need to explain several observed aspects, such as the appearance of a new TL peak at 180 °C in samples doped with Sm^3+^, the single Sm^3+^emission for the low temperature TL peaks and the simultaneous Ce^3+^ and Sm^3+^ emission for the 180 °C TL peak. Besides, the models need to explain the observed fading behavior, which includes the rapid fading rate in the first hours following irradiation.

We speculate that the TL/OSL mechanism in MgO:Li,Ce,Sm possibly involves more than one type of trap and recombination center. During irradiation, electrons and holes are created and are free to move inside the solid. Ce^3+^ traps a hole becoming Ce^4+^, while Sm^3+^ traps an electron becoming Sm^2+^. In fact, the ability of Ce^3+^ to stabilize as Ce^4+^ by trapping a hole and the efficiency of Sm^3+^ as an electron trap has been reported in several studies^34^,^46^,^47^. The reactions during irradiation can be summarized in Equation 1.


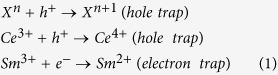


Notice that we have added an unknown *X*^*n*^*-*center, which should act as a hole-trap with stability of a few hours. Examples could be V-type centers, the simplest of which consists of a hole trapped at the Mg^2+^ ion vacancy^28^. A table containing several kind of V-type centers in MgO can be found in Las *et al.*^28^. Curiously, except for V^−^-centers, all listed V-type centers have stability of a few hours. Li is omitted from this equation, but it will be briefly discussed later.

During TL readout, shallow trapped-holes are initially released from *X*^*n+*1^. The freed holes may recombine in Sm^2+^-center (Sm^3+^ + trapped electron) giving rise to Sm^3+^ in the excited state, represented by a star (*) and leading to Sm^3+^ emission during the relaxation process. This mechanism explains the emission spectrum of Sm^3+^ when monitoring the 117 °C TL peak and the Sm^3+^ emission associated with the afterglow (Fig. 6).

It is worth pointing out that this reaction should decrease the amount of Sm^2+^ in the same amount of freed holes from *X*^*n+*1^, reducing the amount of Sm^2+^ defects (trapped electrons) available. This is equivalent to depopulating the Sm^2+^ electron traps even without sufficient thermal energy to release the trapped electrons in Sm^2+^-centers. This may explain the high fading rate observed in the first hours following irradiation in this sample, which is due to the recombination of a freed hole, released from a shallow hole-trap, in a Sm^2+^ -center. As a result, one observes the Sm^3+^ emission spectrum (Fig. 7).


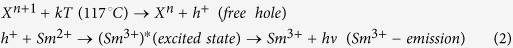


Increasing the reading temperature, an electron trapped at Sm^2+^-center has now sufficient thermal energy (*kT*) to escape. The freed electron recombines with Ce^4+^ turning it into Ce^3+^ in the excited state. By relaxing, this compound emits the characteristic Ce^3+^ emission spectrum. Equation 3 summarizes these mechanisms.


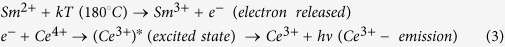


This mechanism explains the TL peak at 180 °C obtained by monitoring the Ce^3+^ emission spectrum (Fig. 7). Nevertheless, by monitoring one of the lines of Sm^3+^ spectrum (578 nm) one can note that this emission also is present in the TL peak at 180 °C (Fig. 7). This could erroneously lead to the conclusion that Sm^3+^ acts also as a recombination center at this temperature. In fact, the Sm^3+^ emission occurring in 180 °C TL peak is probably due to energy transfer from Ce^3+^ to Sm^3+^ defects. The Ce^3+^ to Sm^3+^ energy transfer is favorable by the strong overlapping of the emission spectrum of Ce^3+^ with the absorption spectrum of Sm^3+^ 40. Notice that the reverse, i.e., energy transfer from Sm^3+^ to Ce^3+^, did not happen. The fact that Ce^3+^ has allowed transition increases the efficiency of energy transfer^8^. What has been discussed is also true for the OSL mechanism, since the OSL signal is originating from the trapping centers associated with the 180 °C TL peak (Fig. 5).

**Figure 11:**
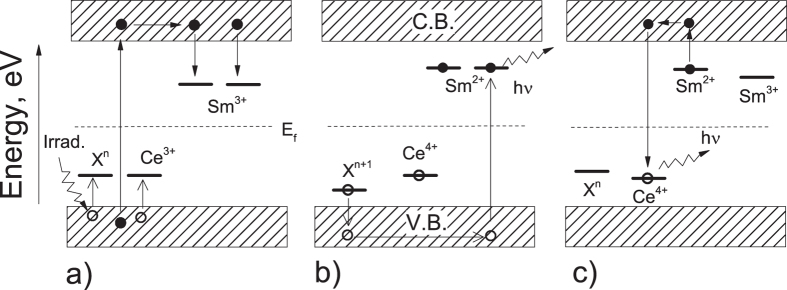
Schematic representation of the proposed model for TL mechanism: (**a**) ionization (excitation across the band gap) and trapping (charges of different sign are trapped by electron- and hole-traps); (**b**) radiative recombination at Sm^2+^-site due to escape of trapped hole from shallow trap at room temperature; and (**c**) radiative recombination and the emission of light. Electrons, solid circle; holes, open circles. Electron transitions, solid arrow; hole transitions, open arrow. E_f_ stands for fermi level.

The proposed model for TL/OSL mechanism is summarized in Fig. 11. (a) The absorption of ionizing radiation results in the ionization of valence electrons, producing free electrons (solid circles) in the conduction band and free holes (open circles) in the valence band. Electrons are free to move in the conduction band and holes, in the valence band. Occasionally, electrons can be trapped by electron-trap and holes by hole-trap defects. (b) During storage immediately following irradiation some of the holes in X can escape at room temperature, recombining with the electrons trapped at Sm sites. (c) Under thermal stimulation (or optical) charges are released from these traps and may recombine in a defect of opposite sign resulting in the emission of light characteristic of that defect. In the figure, the electron-traps (acceptors) are situated above the Fermi level (E_f_) and the hole-traps (donors) below the Fermi level.

It was shown that the Li doping did not changed the TL curve shape of MgO:Ce,Sm, but it enhanced the TL intensity. It has been shown that co-doping MgO:Ln (with Ln = Ce, Eu, Dy) with Li enhanced the RL intensity in order of magnitude^32^,^48^. Apparently, Li plays several roles, including charge compensation and possibly lattice stress relieve on lanthanides doped MgO, due to its reduced ionic radius. The ionic radius of lanthanides is much larger than that of Mg^2+^. This will induce strong distortion and strain around these ions, which will have an influence even far from them^45^.

## Conclusions

In this work, relevant dosimetric, luminescent and morphologic properties of the newly developed OSL/TL material based on triply doped MgO:Li,Ce,Sm were reported and discussed for the first time.

It was demonstrated that doping MgO with Sm^3+^ creates a new TL peak at 180 °C, which is enhanced by introducing a small amount of Ce^3+^ that acts as a luminescent center. Further enhancement was obtained by doping MgO:Ce,Sm with at least 3% Li co-doping (in mol). The starting material purity and the annealing temperature have a major influence in the TL/OSL signal intensity, but not in the shape of the TL/OSL curves.

The triply doped MgO showed an ultra-high sensitivity to ionizing irradiation and wide dynamic range. The calculated minimum detectable dose using the total OSL curve and the initial intensity were 7 μGy and 0.2 μGy, respectively. The detector was able to respond linearly to a wide dynamic range (2 mGy to 20 Gy). The OSL signal showed fading of 6–7% during the investigated period of 35 days, which is consistent with the kinetics of the trapping center, although we cannot exclude the influence of the dim light during detector handling. It was demonstrated that a high batch-to-batch repeatability (10% standard deviation) can be achieved using the SCS method.

In summary, this new phosphor shows suitable dosimetric properties. Besides that, the fast luminescence center associated with Ce^3+^ emission, the possibility of stimulating the sample in the range of infrared to red light, and the spherical and small size of the crystallites (5 μm) supports the potential application of this material for 2D dosimetry, although the observed fading must be considered.

A model was also proposed to explain the basic luminescence properties of this material.

## Additional Information

**How to cite this article**: Oliveira, L. C. *et al.* MgO:Li,Ce,Sm as a high-sensitivity material for Optically Stimulated Luminescence dosimetry. *Sci. Rep.* **6**, 24348; doi: 10.1038/srep24348 (2016).
